# The Value of Active Co-Operation by Civilians

**Published:** 1914-10-17

**Authors:** 


### THE MAKING OF A TERRITORIAL HOSPITAL.

### The Value of Active Co-operation by Civilians.

The interest of ordinary members of the public is not unnaturally centred in the organisation of the Expeditionary Force and the development of Kitchener's Army. It is well, therefore, that the quiet zeal and enthusiasm of the various units of the hospital and ambulance services at home should be recognised and recorded. The keenest interest in and appreciation of these services must be quickened by the account of the organisation and equipment of the military hospital at Leicester, which is only a type of similar hospitals in the various counties of Great Britain, the story of the establishment of most or all of which we hope to be able to tell through the co-operation of experienced and interested men.

### Reclaiming a Deserted Building.

Mr. Johnson, of the Leicester Royal Infirmary, to whom we are indebted for the account which follows, asks us first to imagine a huge building on an eminence commanding a view of a large portion of the county, occupying thirty-seven acres, formerly used as a lunatic asylum, but for seven years past unoccupied, uncleaned, and uncared for externally and internally. This neglect had caused the plaster of the rooms to crumble with decay. The buildings had been left untouched since they were quitted seven years ago, and there are inches of dust on floors, walls, fittings, and indeed everywhere. The grounds have become a wilderness with masses of overgrowth in luxurious abundance, causing what were once roadways and paths to intermingle with the grass lawns and to become almost indistinguishable from them. Such was the state of affairs on August 5 last when these unpromising materials were hurriedly requisitioned by the Leicestershire and Rutland Territorial Association for the purposes of the 5th Northern General Hospital. The military authorities showed courage and judgment when they commandeered the old lunatic asylum, which have been abundantly justified not only on economic grounds, but by the excellent accommodation * thereby provided for wounded soldiers upon a site which is almost ideal in a Midland county.

There are historic associations connected with this Leicester site. It was, there is little doubt, part of a site granted originally to the burgesses of Leicester for their use and occupation for ever by Simon de Montfort, Earl of Leicester, then Lord High Steward of England, whom historians have described as a prince among administrators, a strong man, versatile, great alike in war and peace, great in faith and love of justice, whilst his government of the town and of his estates was so wise and just that it earned for him the sobriquet of Simon the Righteous, and being the son of one who had become practically master of Southern France by his skill in the Crusades, there is a historic fitness about the selection of the Leicester site for a military hospital to accommodate the wounded soldiers of the allied armies of Britain and France in the present war.

### The Architect's Achievement.

Reverting to the buildings to be converted into a military hospital and their terrible condition of neglect, it will be recognised that it required an architect of ability who had had an unusual and successful experience in hospital construction and hospital requirements to render it possible that such a place could be transformed in six weeks into a military hospital with adequate accommodation, not only for the staff and the patients, but with lavatory provision, ventilation, heating, lighting, an operation-theatre, separate pavilions for the R.A.M.C. *personnel*, a modern kitchen with up-to-date fittings, a telephone system, a mortuary, and innumerable other essentials. To accomplish this entailed great skill, especially as cost was a grave consideration, for the alterations necessarily included the lowering of all the old windows by cutting away some four feet of brickwork, the provision of new windows to ensure the maximum ventilation, and the widening of the staircases to admit the conveyance of patients on stretchers.

In the true spirit of the time Leicester determined to utilise first all the talent of the county that could contribute to a successful result. Mr. S. Perkins Pick, the architect of the Leicester Royal Infirmary, where he has done notable work, who is at present engaged in the reconstruction of and additions to Addenbrooke's Hospital, Cambridge, and other important buildings, was appointed architect. The labour involved kept him busy night and day, and it is simple justice to record the remarkable success which he has achieved in making an old building erected in 1830 suitable for the reception of surgical cases. Without pretending to provide many of the refinements which are to be seen in the modern voluntary hospital, the Territorial Hospital at Leicester contains all the details necessary to the successful administration of a military hospital. Leicester tradesmen co-operated with a will in providing the furniture, including mattresses, bolsters, lockers, and bedding, at a minimum cost. One feature of this military hospital is that it contains a number of pictures, the gifts of various friends, which the commanding officer has allowed to be hung in the wards. He holds that suitable pictures are helpful in a military hospital, and may be encouraged without any risk of evil consequences resulting.

The kitchens are well placed, and enable the meals to be served expeditiously to all portions of the building in separate tins which are placed in heated trolleys for conveyance to the wards. A feature not to be found in a voluntary hospital is the pack store, which contains the patients' service uniforms and other equipments.

* See The Hospital, October 10, 1914, page 33, in which the plans appeared.

The Medical and Nursing Staff.

To the Commandant, Lieut.-Colonel Louis K. Harrison, with the co-operation of Major R. Wallace Henry, who has been appointed on the permanent staff, belongs the distinction of having transformed the building, which a month previously was a mass of cobwebs and dirt, into a fitting and comfortable hospital for the reception of wounded soldiers. The services in this connection of Captain Emerson, R.A.M.C., an experienced and sound administrator, proved invaluable in the early days of the institution. Miss C. E. Vincent, the able and successful matron of the Leicester Royal Infirmary, is the matron-in-chief. She has been fortunate in securing as resident matron to the military hospital Miss Hannath, the matron of the Wolverhampton General Infirmary. Miss Vincent took infinite pains in her selection of the members of the nursing staff to spread the mobilisation orders as equally as possible over the whole of the institutions from which the nurses have been drawn. While testifying to the services of these officers, we must not fail to acknowledge that the organisation of the hospital *personnel* and the efficiency of the hospital services were built up in time of peace by Colonel Astley V. Clarke, Assistant Director of Medical Services, North Midland Division, the first Commandant of the hospital.

Leicester is, and deserves to be, proud of the military hospital in its midst, in the preparation of which so keen an interest has been taken. Recognition is due to the services of Mr. A. W. Faire—one of Leicester's worthies—the County Director and District Commissioner of Voluntary Aid Detachments, who undertakes the organisation for the conveyance to the military hospital of the wounded soldiers on arrival at Leicester Station. So far four ambulance trains have arrived—the first on September 3, when there was one stretcher case, the second on September 20, when there were twenty-five cot cases, many of a serious nature, the third on October 7, and the fourth on October 14. The ambulance train (No. 12) was in charge of Captain Stracey, R.A.M.C., during the journey from Southampton.

The transport of the patients had naturally to be undertaken with great care, and, thanks to the facilities afforded by a fleet of motor-cars which the owners placed at Mr. Faire's disposal at the suggestion of the local Automobile Club, the transfer of the soldiers from the trains to the hospital has been performed most efficiently by the staff, and received the warmest praise from the Surgeon-General of the Northern Command.

A Territorial Hospital's Side Shows.

The organisations for the cheer of the wounded patients are many, but certainly one of the most attractive to the men is the Games and Literature Committee, of which Mrs. L. K. Harrison is the President and Mr. Walter G. Gibbs the Hon. Secretary. This committee has been very successful in its methods, and has arranged for the frequent distribution of games, magazines, tobacco, etc., contributed by many enthusiastic Leicester shopkeepers. This committee has also arranged with the local press for a supply of daily and weekly newspapers. Under the supervision of the chaplain, the Rev. W. Cyril Luxmoore, a series of entertainments has been provided in the mess-room every Tuesday and Saturday evening, and the programmes have included various topical songs, such as the now well-known "It's a long way to Tipperary" and "You'll have to go under, Wilhelm." This series of entertainments has been much enjoyed.

"A Patients' Aid Fund."

The officer commanding has instituted a Patients' Aid Fund, which has been readily responded to by the residents, and upwards of £800 has been raised for the provision of comforts for the patients and nursing staff. This fund is most helpful in many ways to the soldiers, some of whom are without money, and although they are always provided with railway passes to their depôts, need cash for stamps, food on their journeys to the depôts, and many other little necessities.

Incidental Features.

A playing field of three acres has been provided. The hospital grounds are being laid out under the supervision of the architect, the overgrowth of trees and shrubs has been cleared away, walks remade, and garden beds planted with bulbs and cuttings. Patients are permitted to see friends on application in writing to the commanding officer. To co-ordinate all the various agencies for the supply of articles for the soldiers, the Mayoress of Leicester, Mrs. Russell Frears, has been indefatigable in her efforts, and much wasted energy and effort has thereby been guarded against. Throughout the Mayor and Mayoress have been tireless in their support, and their thoughtfulness was shown recently in the presentation of framed steel engravings of the King and Queen.

The Prince of Wales' and County Funds.

No less pleasing is it to record the generous way in which the residents of the town and county of Leicester have responded to the Prince of Wales' appeal for the relief of distress occasioned by the war. The County Fund already amounts to upwards of £8,000, in addition to £2,350 for the Patriotic Fund, while a sum of upwards of £21,700 has been contributed to the Mayor of Leicester's Fund.

